# Standalone methacrylated extracellular matrix for digital light processing bioprinting: a practical workflow

**DOI:** 10.3389/fbioe.2026.1774476

**Published:** 2026-04-14

**Authors:** Hod Bruck, Shachar Sofer, Aharon Lion, Asher Ornoy, Udi Sarig

**Affiliations:** 1 Department of Chemical Engineering, Faculty of Engineering, Ariel University, Ariel, Israel; 2 The Department of Morphological Sciences and Teratology, The Dr. Miriam and Sheldon Adelson School of Medicine, Ariel University, Ariel, Israel; 3 Department of Medical Neurobiology, Faculty of Medicine, The Hebrew University of Jerusalem, Jerusalem, Israel

**Keywords:** acellular printed constructs, digital light processing (DLP), lithiumphenyl-2,4,6-trimethylbenzoylphosphinate (LAP), methacrylated decellularized extracellular matrix (dECM-MA), tartrazine, uterine extracellular matrix model, vat photopolymerization (VPP)

## Abstract

Decellularized extracellular matrix (dECM) materials are widely reported to present tissue-specific biochemical cues that influence cell behavior; here, we use porcine uterine dECM as a representative soft-tissue model to operationalize a practical digital light printing workflow. Digital light processing (DLP) offers high-fidelity, photopolymer-based fabrication that avoids shear stresses associated with extrusion and enables precise layer definition, yet its application to soft-tissue dECM remains limited. We produce and evaluate a standalone methacrylated dECM (dECM-MA) formulation and a stepwise, reproducible recipe using lithium phenyl-2,4,6-trimethylbenzoylphosphinate (LAP) and tartrazine. Histological and biochemical analyses confirm successful decellularization, substantial collagen retention, partial sGAG retention, and controlled methacrylation of accessible primary amine groups. The formulation prints via DLP to yield reproducibly defined acellular constructs at a 50-µm layer height and millimeter-scale geometries, demonstrating high dimensional fidelity and controlled swelling behavior. High resolution scanning electron microscopy (HR-SEM) imaging of printed ECM-MA and solid decellularized uterine tissue demonstrated no significant differences in porosity, pore size distribution profiles, and connectivity, as quantified by image analysis, suggesting similar capacity to support diffusion and cell penetration. *In vitro* studies with human uterine stromal fibroblasts—parenchymal cells of the endometrium—show surface attachment and early cell–matrix interaction on printed constructs. Together, these results establish a practical 405-nm digital light printing workflow for standalone methacrylated dECM, exemplified using uterine ECM, enabling acellular construct stereolithographic fabrication with preserved ECM features and compatibility with early cell attachment and histological processing.

## 1 Introduction

Decellularized extracellular matrix (dECM) bioinks have become central to tissue engineering, as they retain tissue-associated extracellular components and structural motifs that have been widely reported to influence cell adhesion, proliferation, and lineage-specific behavior in a tissue-dependent manner (Ramos-Rodriguez and Leach, 2025). Indeed, the ability of dECM bioinks to recapitulate the complex microenvironment of native tissues underpins their widespread adoption in regenerative medicine (Wang et al., 2024) and advanced 3D bioprinting applications (Elomaa et al., 2023).

Within the wider context of biomaterials and tissue engineering, the evolution of 3D bioprinting modalities (Wu et al., 2023) reflects ongoing efforts to balance resolution, material versatility, and bioactivity (Cabral et al., 2024). For instance, inkjet molecular patterning (an early form of inkjet printing) introduced in the late 1980s (Klebe, 1988) matured in the early 2000s with the first demonstration of viable cell-patterning (Boland et al., 2003; Wilson and Boland, 2003). This approach offers high-resolution bioprinting (20–100 μm) and gentle handling of cells (Huang et al., 2024), but is limited to low-viscosity bioinks (3–30 mPa·s) (Zhao et al., 2023) and often requires synthetic cross linkers for stabilization (Liu and Wang, 2020; Lai et al., 2024), which can compromise mechanical integrity and restrict the use of complex, tissue-mimetic materials (Budharaju et al., 2024). Extrusion-based bioprinting, which became prominent in the 2010s, enabled the dispensing of high-viscosity shear-thinning bioinks (Zhao et al., 2021; Elomaa et al., 2023) including dECM (Zhang H. et al., 2023), while also allowing multimaterial deposition for heterogeneous tissue constructs (Betancourt and Chen, 2022). However, extrusion sacrifices resolution (typically >200 μm) (McLoughlin et al., 2024), exposes cells to shear stress (Gharraei et al., 2024; Huang et al., 2024), and often necessitates blending dECM with polymers such as gelatin methacryloyl (GelMA) (Zhang M. et al., 2023) or hyaluronic acid methacrylate (HAMA) (Bebiano et al., 2023) to optimize printability and stability (Li et al., 2023), which can dilute native ECM components (Xu et al., 2024). Volumetric bioprinting has more recently enabled rapid fabrication of entire 3D constructs using visible-light crosslinking systems like ruthenium/sodium persulfate (Ru/SPS) for standalone dECM bioinks (Lian et al., 2024). Yet, volumetric approaches are limited by relatively coarse voxel resolution (∼300 μm) (Antoine et al., 2023) and high bioink consumption (>5 mL/construct) (Krumins et al., 2024), restricting scalability (Krumins et al., 2024) and fidelity for microscale tissue features (Madrid-Wolff et al., 2023).

Digital light processing (DLP) balances these trade-offs by enabling precise layer bioprinting (∼50 μm) and pixel-defined in-plane resolution through simultaneous exposure of entire layers, resulting in rapid, layer-by-layer fabrication (Jeong et al., 2024) that is ∼20 times faster than extrusion based printing. These features make DLP an attractive platform for high-fidelity soft hydrogel fabrication (Grigoryan et al., 2019; Kim and Cho, 2024), yet its application to standalone soft-tissue dECM remains limited. Thus, comprehensive reviews have summarized chemical and processing strategies to improve the printability of dECM-based bioinks, e.g., (Zhang H. et al., 2023). These efforts, however, predominantly address extrusion-based or volumetric approaches, and reported DLP-compatible formulations generally rely on hybrid systems incorporating synthetic polymers such as gelatin methacryloyl (GelMA) or polyethylene glycol diacrylate (PEGDA) (Lian et al., 2024; Alparslan and Bayraktar, 2025). Of note is that both PEGDA and GelMA lack the biochemical complexity of native ECM (Bae et al., 2024). While methacrylation improves photocrosslinking kinetics of dECM under visible light, existing dECM-MA formulations often require blending with additional polymers or face slow curing (>60 s radiation/layer) compromising bioactivity and print fidelity (Almalla et al., 2024). Furthermore, recent reports of standalone dECM bioinks crosslinked with Ru/SPS, successful in volumetric bioprinting (Lian et al., 2024), remain incompatible with DLP due to differences in optimal crosslinking wavelengths (450–500 nm vs. DLP’s 405 nm) (Jeong et al., 2024) and oxygen inhibition occurring in thin layers’ bioprinting (Lai et al., 2024). This persistent gap underscores the need for standalone, rapidly crosslinkable dECM bioinks optimized for high-resolution DLP bioprinting (Lai et al., 2024).

This work introduces a standalone methacrylated dECM bioink optimized for DLP using lithium phenyl-2,4,6-trimethylbenzoylphosphinate (LAP) and tartrazine, enabling rapid photocrosslinking (8–30 s/layer) while preserving structural and compositional features of tissue-derived dECM that, according to prior literature, is associated with tissue-specific bioactivity. By leveraging tartrazine’s photoabsorptive optical attenuation to confine photopolymerization in thin layers (He et al., 2023) and LAP’s 405 nm compatibility (Yang et al., 2024), we achieve ∼50 μm resolution without synthetic additives, advancing toward functional regenerative therapies and other biomedical applications.

## 2 Materials and methods

### 2.1 Porcine uteri decellularization

Porcine uteri, used here as a model barrier soft tissue, were obtained from 3–5-month-old gilts through the Lahav Animal Research Institute (Lahav, Israel). Upon isolation, uteri were placed in ice-cold phosphate-buffered saline (PBS) supplemented with antibiotics (100 U/mL penicillin and 100 μg/mL streptomycin) and transported for same-day processing. Uterine horns were cleaned of excess fascia and residual peritoneal tissue, then dissected and sectioned under sterile conditions into uniform 1 × 1 cm fragments.

Tissue fragments underwent four alternating cycles of immersion in hypertonic (10% NaCl) and hypotonic (distilled water) solutions, each for 20 min, to promote osmotic disruption of cellular membranes. Subsequently, samples were incubated in 1% Triton X-100 for 48 h, followed by 1% sodium dodecyl sulfate (SDS) for 2 × 48 h cycles and an additional 24 h SDS cycle with fresh solutions in each cycle under continuous agitation at room temperature. Residual detergents were removed by washing for 5 min under flowing tap water, followed by a 5-min wash in large-volume PBS. The decellularized tissues were then stored in PBS at 4 °C until further processing or lyophilization of frozen ECM samples in distilled water filled tubes.

### 2.2 ECM liquefaction and chemical modification

Decellularized uterine tissue was frozen in distilled water at −80 °C, immersed in liquid nitrogen for 10 min and lyophilized until completion using a freeze dryer (Labconco Corporation, Kansas City, MO). Lyophilized samples were milled into a fine powder using a bead mill homogenizer equipped with 2.8 mm beads (Omni Bead Ruptor, Omni International Inc., Kennesaw, GA). For homogenization, approximately each 250 mg of lyophilized ECM was inserted into a single 15 mL tube with 5 g of ceramic beads and allowed to homogenize in up to ten cycles of 1 min each. Pauses between cycles were made to assure ECM temperature does not increase above 37 °C, as verified by a digital thermometer. The resulting extracellular matrix (ECM) powder was passed through a 0.4 mm mesh sieve to ensure particle uniformity.

To liquefy ECM components the ECM powder was digested in 0.1 M hydrochloric acid (HCl) containing 15% (w/w) pepsin:ECM ratio (Pepsin from gastric mucosa, 1,200–1400 U/mg, Sigma-Aldrich, St. Louis, MO) at a concentration of 35 mg ECM/mL digestion solution under gentle stirring at room temperature for 60 h. Following digestion, the pH of the solution was adjusted to 9.6 by potentiometric titration with NaOH 1M to terminate enzymatic activity directly followed by methacrylation (∼10% additional volume due to NaOH addition). Methacrylation was performed by drop-wise addition of 2.5% (v/v) methacrylic anhydride (Sigma-Aldrich, St. Louis, MO) at 4 °C under strict pH monitoring and continuous stirring for overnight incubation. Excess reagents and reaction byproducts were removed via dialysis against 0.02 M sodium phosphate buffer (Na_2_HPO_4_, pH 7.4) using 12 kDa molecular weight cutoff (MWCO) dialysis tubes (Spectrum Laboratories Inc., Rancho Dominguez, CA) for 72 h, with buffer changes every 24 h. The final methacrylated ECM (ECM-MA) product was lyophilized and stored at −20 °C under desiccator conditions until further use.

### 2.3 dECM bioink formulation and DLP bioprinting

To optimize the bioink formulation for DLP bioprinting, various concentrations of processed dECM powder, photoinitiators, and photoabsorbers were evaluated. Specifically, combinations of LAP with tartrazine, and a ruthenium-based system comprising tris(2,2′-bipyridyl) dichlororuthenium (II) hexahydrate and sodium persulfate (Ru/SPS; Ruthenium Visible Light Photocrosslinking Kit, Cat. No. 5248-1KIT, Advanced BioMatrix, Carlsbad, CA, United States) were tested. However, formulations utilizing the Ru/SPS system did not yield satisfactory bioprinted constructs under the applied DLP bioprinting conditions (Supplementary Figure S1 and Supplementary Material Online). Among the LAP-tartrazine formulations, the composition detailed below produced constructs with optimal handling characteristics for the specific ECM source and animal age while minimizing component concentrations.

The DLP standalone dECM bioink formulation was prepared by dissolving 8%–10% (w/v) methacrylated dECM (ECM-MA) in phosphate-buffered saline (PBS) containing 0.5% (w/v) LAP (Sigma-Aldrich, St. Louis, MO, United States) and 1.5 mM (0.08% w/v) tartrazine (Merck KGaA, Darmstadt, Germany). The solution was homogenized until uniform using a benchtop homogenizer (Model HOG-160-1; MRC Ltd., Holon, Israel). Approximately 300 µL of the bioink was loaded into a Lumen X DLP bioprinter (CELLINK, Gothenburg, Sweden). Constructs were printed with a layer thickness of 50 μm, using 405 nm light at 80% intensity, with an exposure time of up to 30 s per layer. The first layer received four times the standard exposure to ensure proper stage adhesion. A standard cylindrical geometry (8 mm diameter, 3 mm height) was fabricated to assess printing fidelity.

### 2.4 Bioprinted dECM construct characterization

dECM constructs were characterized by quantifying total collagen, double-stranded DNA (dsDNA), and sulfated glycosaminoglycan (sGAG) content. Lyophilized native porcine uterine tissue and decellularized porcine uterine tissue served as positive and negative controls, respectively. Each experimental group comprised 12 independent samples (n = 12) derived out of four independent ECM productions (a triplicate per ECM uterus source), providing sufficient statistical power for comparative analysis.

#### 2.4.1 Collagen quantification

Total collagen content was measured using the Hydroxyproline assay as previously reported (Samuel, 2009). Briefly, samples were hydrolyzed in 6 M hydrochloric acid at 110 °C for 24 h, followed by a colorimetric reaction with 4-(dimethylamino) benzaldehyde (DMAB). Absorbance was measured at 558 nm using an Infinite M Plex multimode microplate reader (Tecan Group Ltd., Seestrasse 103, 8708 Männedorf, Switzerland). Collagen concentration was calculated based on a hydroxyproline standard curve prepared using trans-4-hydroxy-L-proline (Sigma-Aldrich; Cat. No. 41875-100MG), assuming hydroxyproline constitutes approximately 14.4% of total collagen by weight.

#### 2.4.2 DNA quantification

Residual dsDNA content was assessed using the Quant-iT™ PicoGreen® dsDNA Assay Kit (Thermo Fisher Scientific, Waltham, MA, United States) as previously described (Hoemann et al., 2002). Samples were incubated with the PicoGreen reagent, and fluorescence was measured with excitation at 480 nm and emission at 520 nm using the same microplate reader. DNA concentrations were determined by comparison to a standard curve generated with lambda DNA standards provided in the kit.

#### 2.4.3 GAG quantification

sGAG content was quantified using the dimethylmethylene blue (DMMB) assay, as previously described (Hoemann et al., 2002; Coulson-Thomas and Gesteira, 2014). Briefly, samples were reacted with DMMB dye solution (1,9-Dimethyl-Methylene Blue zinc chloride double salt; dye content 80%; Sigma-Aldrich, St. Louis, MO, United States; Cat. No. 341088-1G), and absorbance was read at 525 nm using the Infinite M Plex microplate reader. Chondroitin sulfate (Sigma-Aldrich, St. Louis, MO, United States) was used to generate the standard curve for quantification.

#### 2.4.4 Degree of methacrylation quantification (TNBS assay)

The degree of methacrylation of ECM-MA was quantified using a 2,4,6-trinitrobenzenesulfonic acid (TNBS, Sigma-Aldrich, St. Louis, MO, United States; Cat. No. P2297) assay to determine the reduction in accessible free primary amine groups following methacrylation. Lyophilized dECM and ECM-MA were dissolved in 0.1 M sodium bicarbonate buffer (pH 8.5) at a concentration of 5 mg mL^-1^. For each sample, 85 µL of polymer solution was mixed with 85 µL of TNBS reagent and incubated for 2 h at room temperature. The reaction was quenched by addition of 42.5 µL of 1 M hydrochloric acid (HCl), followed by 85 µL of 10% (w/v) SDS. Absorbance at 335 nm was measured using a microplate reader (Infinite 200, Tecan). A calibration curve generated using Glycine standards was used to convert absorbance values to the mass of accessible primary amines. The degree of methacrylation (%) was calculated by comparing the amine content per unit mass of ECM-MA to that of unmodified dECM, expressed as the percentage reduction in amine/ECM weight ratio (mg/g) relative to native dECM. Measurements were performed in triplicate from independent ECM preparations unless otherwise stated.

### 2.5 Bioink printability assessment

To quantitatively evaluate bioink printability under DLP conditions, standardized cubic constructs (5 × 5 × 5 mm) were fabricated using identical printing parameters (50 µm layer thickness, 405 nm illumination at 80% intensity, ≤30 s exposure per layer). Methacrylated dECM bioink (8% w/v ECM-MA; n = 3 independent prints) and PEGDA control constructs (10% w/v PEGDA; n = 5 independent prints) were prepared and printed under identical photopolymerization conditions. Immediately after printing and gentle PBS rinsing, constructs remained transiently anchored to the build stage, indicating effective layer-by-layer crosslinking. Cubes were detached and edge lengths were measured using a calibrated digital caliper to assess dimensional fidelity relative to the 5 mm CAD design. To evaluate post-print structural stability, constructs were incubated in PBS at 37 °C for 48 h and re-measured. Linear swelling was calculated as a length (L) percentage based on the following formula: [(L_48h_ − L_0_)/L_0_] × 100, providing a direct indicator of dimensional stability following incubation. Data are presented as mean ± SD, with individual data points plotted to reflect inter-print variability.

### 2.6 Histology and scanning electron microscopy (SEM) imaging

#### 2.6.1 Histology

Native, decellularized, and 3D DLP bioprinted tissue samples were fixed in 4% paraformaldehyde (PFA) for 30 min at 4 °C, followed by thorough washing in phosphate-buffered saline (PBS). Subsequently, samples were dehydrated, paraffin embedded, and sectioned at 5–10 µm thickness. Sections were stained using the Trichrome Stain Kit (Modified Masson’s, ScyTek Laboratories, Inc., Logan, UT, United States; Cat. No. TRM-2), following the manufacturer’s protocol (TRM-IFU). This staining method differentiates tissue components by staining collagen fibers blue, cell cytoplasms in red, and nuclei dark red to black. Stained sections were examined under a light microscope to assess tissue architecture and collagen distribution. Representative images are shown out of at least 2 sections per sample and at least three samples per group.

#### 2.6.2 Scanning electron microscopy (SEM)

For ultrastructural analysis, samples were fixed in 2.5% glutaraldehyde, dehydrated through a graded ethanol series, and sputter-coated with gold to enhance conductivity. Imaging was performed using a TESCAN MIRA3 field emission scanning electron microscope (Tescan Orsay Holding a.s., Brno, Czech Republic), which offers high-resolution imaging capabilities suitable for detailed surface morphology studies.

#### 2.6.3 Quantitative image analysis

SEM images of decellularized tissue and printed ECM-MA constructs were analyzed using Fiji (ImageJ), an open-source image analysis platform (Schindelin et al., 2012). Images were converted to 8-bit grayscale and preprocessed using background subtraction and Gaussian filtering to reduce charging artifacts and high-frequency noise. Binary pore masks were generated by thresholding using the Otsu method, followed by morphological operations (hole filling, opening/closing, and watershed segmentation) to separate adjacent pores. Segmentation accuracy was verified by visual overlay of pore boundaries onto the original SEM images. Quantitative analysis was performed on multiple independent and randomly selected regions of interest (ROIs; n = 5 for decellularized tissue and n = 6 for printed ECM-MA). Two-dimensional porosity was calculated for each ROI as the pore area fraction. Nearest-neighbor pore spacing was computed as the minimum Euclidean distances between pore centroid coordinates. Pore network connectivity was assessed using the MorphoLibJ plugin, and the largest connected pore fraction (LCPF) was defined as the fraction of total pore area belonging to the largest connected pore cluster. Data are reported as mean ± SEM, and group comparisons were performed using unpaired two-tailed t-tests.

Pore size was quantified using the maximum Feret diameter. Pore size distributions exhibited bimodality, and two pore populations were defined using a cutoff at 2 µm. For distribution analysis and curve fitting, pores from all ROIs within each condition were pooled to generate population-level histograms. Each pore population was fitted with a lognormal distribution, and goodness-of-fit was assessed using adjusted R^2^. For comparative analysis of mean pore size between decellularized tissue and ECM-MA, pooled pore populations were used, with outliers excluded prior to analysis using the ROUT (robust regression and outlier removal) method implemented in GraphPad Prism, with a false discovery rate threshold of Q = 1% (Motulsky and Brown, 2006) according to predefined criteria prior to statistical comparison.

### 2.7 Cell culture and phenotypic characterization

Primary human endometrial stromal fibroblasts (hESFs, passage 4) were commercially obtained from Lifeline Cell Technology (Frederick, MD, United States; Cat. No. FC-0076) and cultured using the manufacturer supplied culture media and guidelines. No patient samples or animal subjects were involved. While fibroblasts are typically regarded as stromal support cells in most organs, uterine endometrial stromal fibroblasts perform parenchymal functions by driving the cyclical remodeling of the endometrium and its decidualization – processes essential for the menstrual/reproductive cycle and for embryo implantation (Gellersen and Brosens, 2014).

For phenotypic characterization, cells were fixed on round coverslips with 4% paraformaldehyde (PFA) for 30 min at 4 °C, followed by phosphate-buffered saline (PBS) washes. Immunofluorescence staining was performed to assess the expression of stromal markers (CD73 and vimentin), and the endometrial marker CD10, with CD31 serving as a negative endothelial marker (isotype negative control). The staining protocol involved incubation with primary antibodies at a 1:100 dilution and secondary antibodies at a 1:250 dilution. Specifically, rabbit anti-human CD10 (Bioss, Cat. No. BS-0527R-20; RID: AB_10854297) and rabbit anti-human vimentin (Bioss, Cat. No. BS-0756R-20; RRID: AB_10855343) were used as primary antibodies, with a rabbit IgG isotype control (Santa Cruz Biotechnology, Cat. No. sc-8306; RRID: AB_653100). Secondary detection employed goat anti-rabbit IgG Alexa Fluor 488 (Abcam, Cambridge, United Kingdom; Cat. No. ab150081; RRID: AB_2734747) and donkey anti-rabbit IgG phycoerythrin (PE; Santa Cruz Biotechnology; Cat. No. sc-3745; RRID: AB_641183), the latter used specifically for vimentin. Additionally, a FITC-conjugated anti-CD73 primary antibody (BioLegend, San Diego, CA, United States; Cat. No. 344015; RRID: AB_2561808) was utilized at a 1:50 dilution. Nuclei were counterstained using Fluoromount-G with DAPI (SouthernBiotech, Birmingham, AL, United States). Cells at passage numbers less than 8 (P < 8) were used for all characterization and dECM experiments to ensure cellular consistency.

### 2.8 Bioprinted dECM cell support-ability

dECM concaved cylindrical constructs, each measuring 4 mm in diameter, were prepared for cell seeding experiments. Each construct (n = 3) was seeded with 3.3 × 10^5^ hESFs suspended in 500 µL of culture medium. The constructs were incubated at 37 °C in a CO_2_ incubator for 60 min to facilitate initial cell adhesion. This seeding protocol corresponds to an approximate surface seeding density of 2.6 × 10^6^ cells/cm^2^ (within the same order of magnitude as physiological cell densities).

Post-incubation, the constructs were aseptically transferred to individual wells of a 12-well plate, each containing 2 mL of the same culture medium. The constructs were maintained under static culture conditions for 16 h. Following the culture period, samples were fixed and processed for scanning electron microscopy (SEM) imaging as described above to study surviving cell density and initial cellular remodeling and interaction with the bioprinted dECM.

### 2.9 Ethics statement

Porcine uterine tissues were obtained post-mortem through the Lahav Animal Research Institute (Lahav, Israel). No animal procedures were performed specifically for this study, and therefore institutional animal ethics approval was not required. Primary human endometrial stromal fibroblasts were commercially obtained from Lifeline Cell Technology; the supplier certifies donor consent and de-identification, and no human subjects were directly involved in this research.

### 2.10 Statistical analysis

All experiments were conducted with a minimum of three independent replicates, unless specified otherwise. Data were assessed for normality/lognormality and the absence of outliers prior to analysis. Comparisons between group means were performed using unpaired two-tailed Student’s t-tests, as appropriate, utilizing GraphPad Prism version 9 (GraphPad Software, San Diego, CA, United States; RRID: SCR_002798). A p-value of less than 0.05 was considered indicative of statistical significance.

## 3 Results

### 3.1 A modular dECM biofabrication process

**FIGURE 1:**
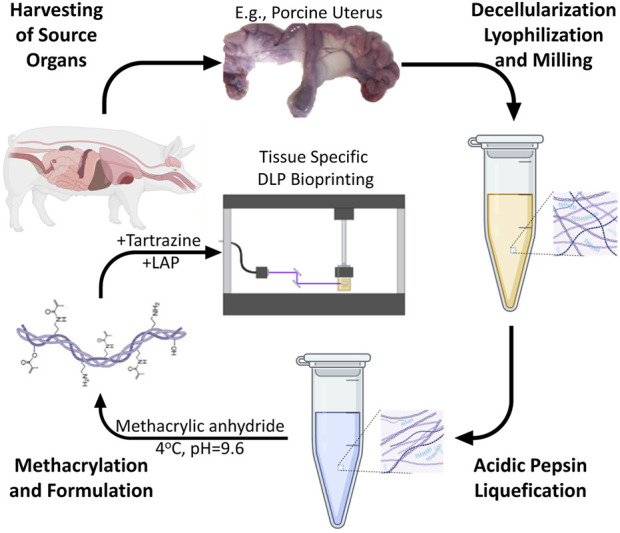
Schematic representation of the tissue derived DLP bioink fabrication protocol consisting of four interconnected phases. The process begins with harvesting source organs (exemplified by porcine uterus), followed by decellularization, lyophilization, and milling to produce homogeneous dECM powder. This powder undergoes acidic pepsin digestion for liquefaction, after which the pH is neutralized. Methacrylation is performed at pH 9.6 using methacrylic anhydride (2.5%) at 4 °C, followed by dialysis and lyophilization. The final bioink formulation combines 8%–10% ECM-MA with 0.5% LAP photoinitiator and 1.5 mM tartrazine photoabsorber. DLP bioprinting is conducted using 405 nm light (80% intensity) with up to 30 s exposure per 50 μm layer, yielding defined 3D constructs with preserved ECM characteristics. Of note is that the process is modular and allows optimization of each phase individually for different tissue sources.

To establish a standalone, ECM bioink derived from a representative soft-tissue source suitable for DLP bioprinting, we developed and validated a stepwise fabrication strategy based on porcine uterine tissue. The process included optimized decellularization, enzymatic liquefaction, methacrylation, and photoinitiator-based formulation compatible with DLP systems. A schematic overview of the entire fabrication pathway is presented in Figure 1, highlighting key phases from organ harvest to final construct printing.

**FIGURE 2:**
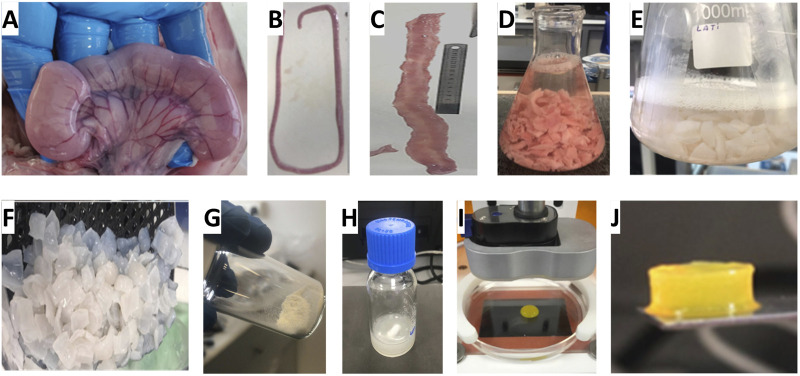
Macroscopic steps in ECM-MA bioink preparation and DLP printing. Porcine uteri **(A)** were dissected to isolate the uterine horns **(B)** after removing excess tissues (peritoneal remnants, fascia, cervix, vagina, oviducts, and ovaries). A spread uterine horn was longitudinally cut (endometrial side up, **(C)** to reveal its tubular structure. The horn was sliced into 1.5–2 cm pieces **(D)** and decellularized **(E)**. The resulting ECM **(F)** was processed into a homogenous ECM-MA powder **(G)**, forming the bioink base after liquefaction **(H)** and methacrylation. The addition of LAP and Tartrazine yielded the final bioink formulation **(I)**, which was used to produce a self-supporting, high-fidelity, disk-shaped construct with clear edges via DLP bioprinting **(J)**.

The porcine uterus was selected as a representative soft tissue model to demonstrate this workflow, owing to its composite architecture comprising epithelial, stromal, and smooth muscle layers—features that are broadly shared across mucosal barrier and organ wall tissues. This diversity in cellular and matrix compartments renders it a valuable exemplar for testing decellularization efficiency, ECM preservation, and compatibility with high-resolution bioprinting. Fresh porcine uteri were harvested from 3–5-month-old gilts, after which the uterine horns were isolated, cleaned of fascia and associated peritoneal structures (Figures 2A,B), and dissected to expose the internal endometrial surface (Figure 2C). The horns were segmented into 1.5–2 cm sections (Figure 2D) and subjected to a refined decellularization protocol involving sequential osmotic, and detergent-based processing (Figure 2E, as detailed in Methods and schematized in Figure 1). The resulting ECM (Figure 2F) was visibly pale and translucent, indicative of efficient removal of cellular components.

### 3.2 dECM bioink formulation and printability assessment

Following lyophilization and bead milling (Figure 2G), the ECM was enzymatically liquefied with acidic pepsin (Figure 2H) and subsequently methacrylated to introduce photocrosslinkable groups. The resulting ECM-MA was combined with photoinitiators and photoabsorbers to produce a bioink compatible with DLP printing. In early optimization experiments, we tested both LAP–tartrazine and ruthenium/sodium persulfate (Ru/SPS) crosslinking systems. However, the Ru/SPS system, despite its utility in volumetric bioprinting, did not yield stable constructs under DLP conditions in our hands and was therefore not pursued further (Supplementary Figure S1 in the Supplementary Material). Instead, LAP–tartrazine combinations demonstrated robust crosslinking under 405 nm exposure and were used in all subsequent printing steps.

Bioink formulations containing 8%–10% ECM-MA, 0.1% LAP, and up to 1.5 mM tartrazine were loaded into the DLP bioprinter (Figure 2I) to fabricate 3D constructs with a standardized cylindrical geometry (Figure 2J). The printed constructs appeared relatively transparent with clearly defined edges. No formal mechanical testing was performed, as the focus of this study was workflow feasibility rather than load-bearing applications. The printed constructs were stable and retained their shape upon routine handling, indicating sufficient integrity for *in vitro* handling and processing.

**FIGURE 3:**
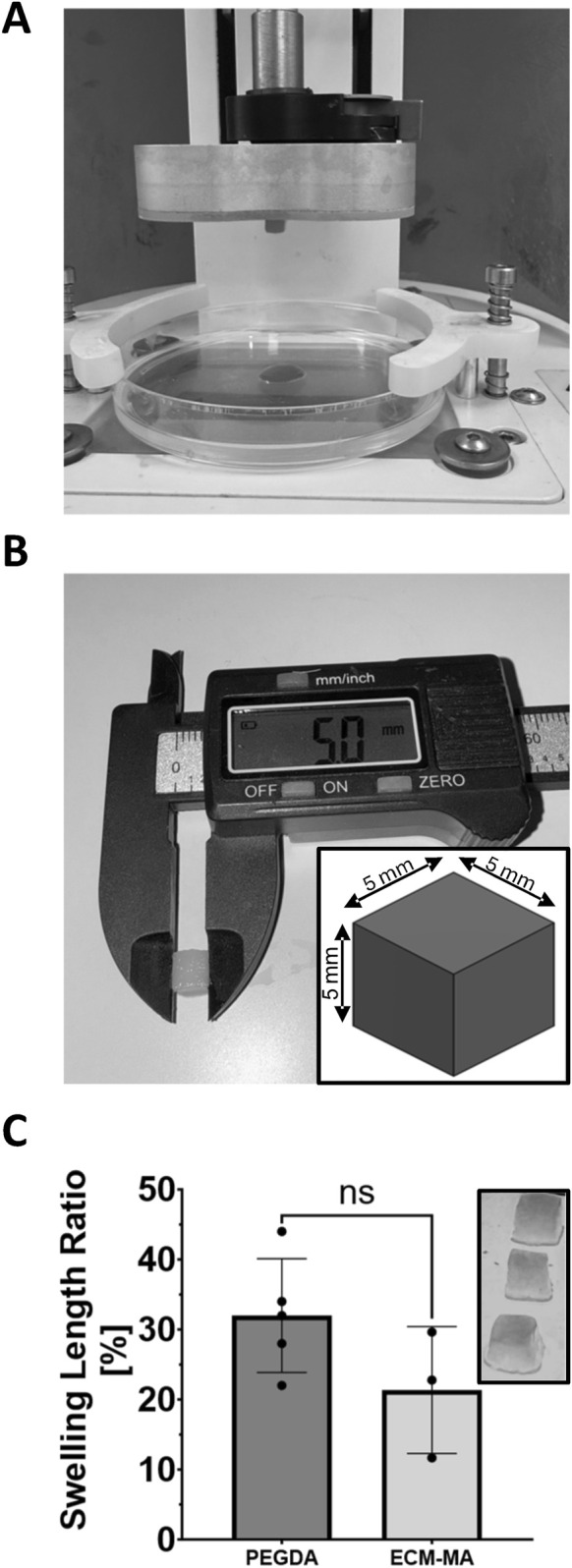
Quantitative assessment of DLP printability, dimensional fidelity, and post-print swelling behavior. Representative image of a 5 × 5 × 5 mm ECM-MA cube immediately following DLP fabrication, transiently anchored to the build stage, demonstrating effective layer-by-layer photopolymerization **(A)**. Digital caliper measurement of the printed construct immediately after detachment, confirming close agreement between the printed edge length and the 5 mm CAD design **(B)**. Linear swelling percentage of printed constructs after 48 h incubation in PBS at 37 °C, calculated as [(L_48h_ − L_0_)/L_0_] × 100 **(C)**. Data are presented as mean ± SD with individual data points shown (8% w/v ECM-MA, n = 3 independent prints; 10% w/v PEGDA, n = 5 independent prints). Although PEGDA exhibited a numerically higher swelling ratio, the difference between groups did not reach statistical significance (unpaired two-tailed t-test, p = 0.1353). Inset: Representative images of triplicate ECM-MA constructs after 48 h incubation, demonstrating reproducible geometric preservation.

To quantitatively assess bioink printability, standardized 5 mm cubic constructs were fabricated and evaluated for dimensional fidelity and swelling behavior (Figures 3A–C). Immediately following printing, cubes exhibited sharply defined edges and remained anchored to the build stage (Figure 3A). Digital caliper measurements confirmed close agreement between printed edge lengths of the ECM-MA cubes and the 5 mm CAD design (Figure 3B), demonstrating high geometric fidelity. After 48 h of incubation in PBS at 37 °C, constructs retained overall cubic architecture with preserved geometric integrity. Linear swelling percentage analysis revealed mean values of 21% ± 9% for 8% ECM-MA (n = 3) and 32% ± 8% for 10% PEGDA (n = 5) (mean ± SD; Figure 3C). Although PEGDA exhibited a numerically higher swelling ratio, the difference between groups did not reach statistical significance (unpaired two-tailed t-test, p = 0.1353). These findings indicate that the standalone dECM-MA formulation demonstrates swelling behavior comparable to a benchmark DLP-printable hydrogel while maintaining reproducible geometric stability.

### 3.3 3D bioprinted dECM characterization

**FIGURE 4:**
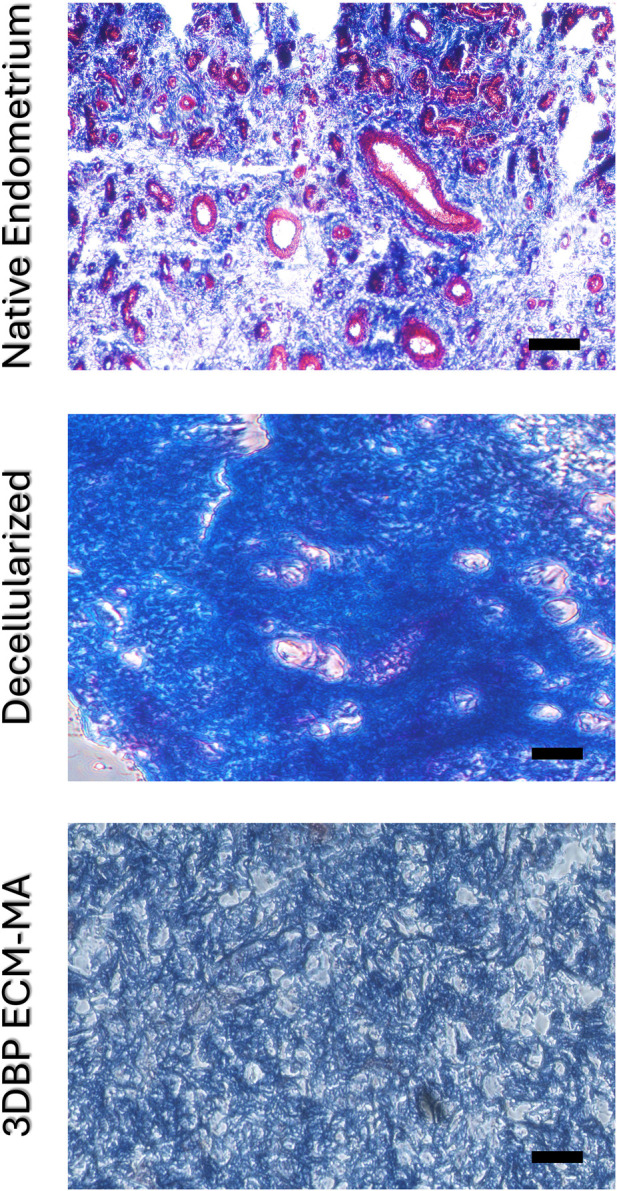
Histological characterization of the 3D DLP bioprinted ECM-MA disk using Masson’s trichrome staining. Staining of the original porcine uterus (top), decellularized tissue (middle), and the printed disk (bottom) reveals the preservation of collagen fiber arrangement and ultrastructure (blue, representing ECM fibers) and significant removal of porcine cellular components (red cytoplasm, black nuclei) in the decellularized tissue and the 3D bioprinted (3DBP) endometrial methacrylated ECM (ECM-MA) construct. Scale bars: 100 μm.

To assess matrix preservation and cell removal, native, decellularized, and 3D-printed samples were histologically stained with Masson’s trichrome (Figure 4). Native uterine tissue exhibited dense red cytoplasmic staining and numerous nuclei, interspersed within blue collagen-rich regions (Figure 4, top). In decellularized samples, cellular staining was virtually absent while the collagen architecture remained intact and prominent (Figure 4, middle). Bioprinted constructs also withstood histological processing (reflecting sufficient handling integrity) and similarly showed blue-stained collagen distribution with no visible nuclear or cytoplasmic residues, indicating that methacrylation and photopolymerization preserved collagen-rich matrix features visible by trichrome staining (Figure 4, bottom).

**FIGURE 5:**
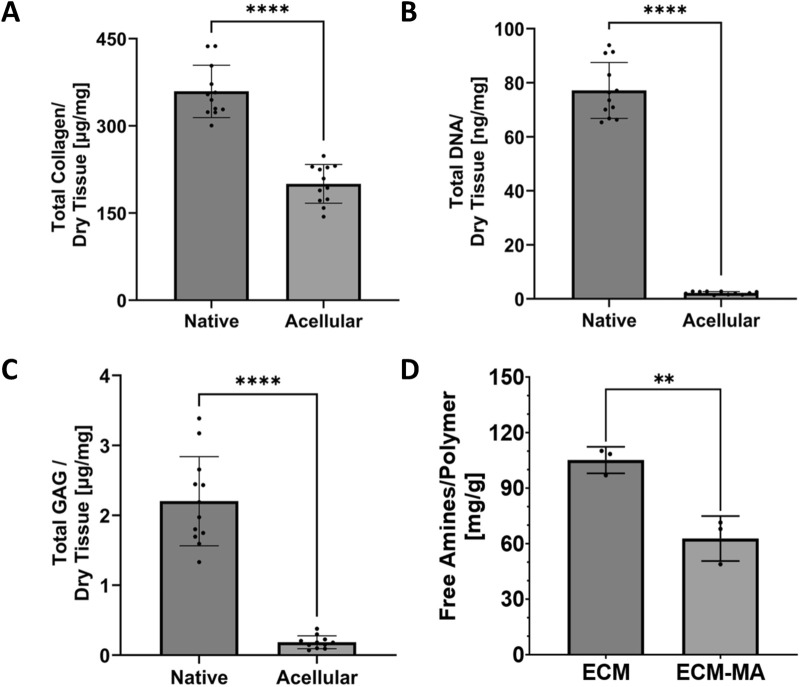
Biochemical analysis of native and decellularized ECM. Collagen content, quantified by hydroxyproline assay, showed over 50% preservation compared to native tissue **(A)**. Collagen, the major structural ECM protein, is crucial for the bioink’s handling stability post printing. DNA quantification **(B)** demonstrated successful removal of cellular content, with less than 50 ng DNA per mg of dry ECM, meeting decellularization standards (Gilbert et al., 2009; Crapo et al., 2011). **(C)** sGAG content, measured using the DMMB assay (Eitan et al., 2010), was significantly reduced post-decellularization. As expected for detergent-based decellularization, sGAG content was markedly reduced, consistent with preferential loss of soluble/loosely bound glycosaminoglycans during processing. Quantification of primary amine groups using the TNBS assay demonstrated a measurable reduction in free amine content following methacrylation, consistent with functionalization of available reactive groups **(D)**. Data are presented as mean ± SD. For panels **(A–C)**, n = 12 per group; for TNBS analysis **(D)**, n = 3 independent preparations. Statistical significance is indicated as follows: **p < 0.01; ****p < 0.0001.

Biochemical analysis further validated matrix preservation. Total collagen content, quantified via hydroxyproline assay, was retained at over 50% of native levels (52.2%) following decellularization (native: 358 ± 48 μg/mg; decellularized: 187 ± 24 μg/mg; p < 0.001, Figure 5A). DNA content dropped from 77 ± 8 ng/mg in the native tissue to 3.0 ± 0.5 ng/mg post-decellularization (Figure 5B), well below the 50 ng/mg decellularization benchmark (Crapo et al., 2011). Sulfated glycosaminoglycans (sGAGs) were partially retained: native levels of 2.2 ± 0.6 μg/mg were reduced to 0.2 ± 0.1 μg/mg after decellularization (p < 0.001, Figure 5C), reflecting the selective loss of soluble or cell surface-associated GAGs during processing. Primary amine content, quantified by TNBS assay, decreased from 105 ± 7 mg/g in dECM to 63 ± 12 mg/g following methacrylation (Figure 5D), corresponding to a 42 mg/g (40%) reduction in accessible amine groups (p < 0.01).

**FIGURE 6:**
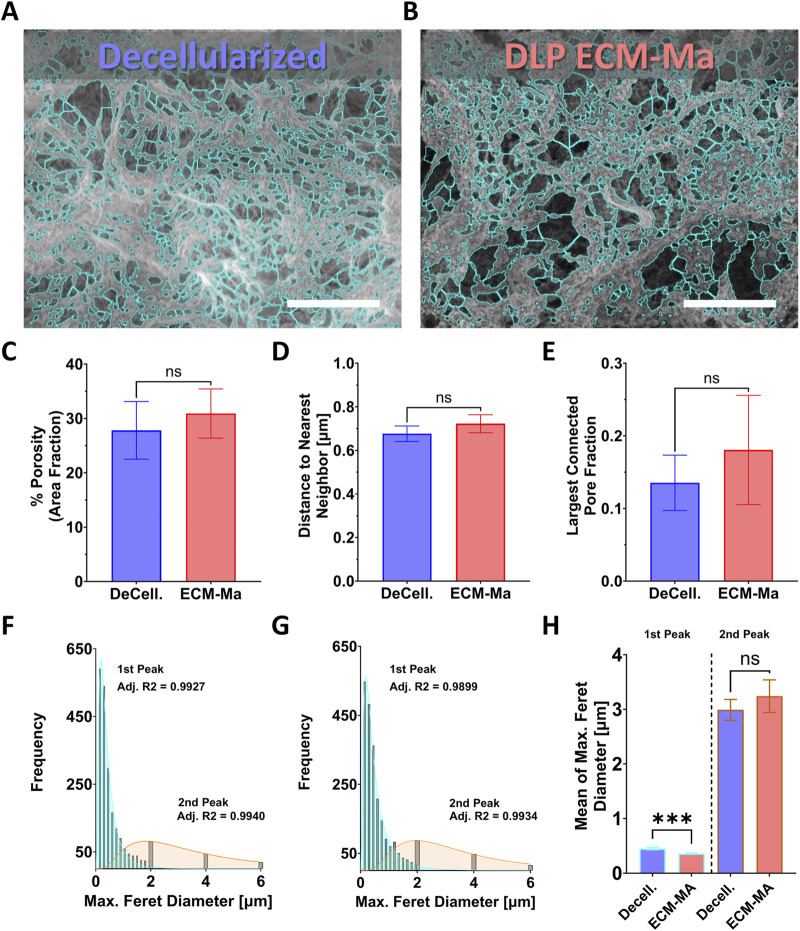
Quantitative ultrastructural comparison of decellularized ECM and DLP-printed ECM-MA constructs. Representative SEM micrographs of decellularized solid uterine ECM **(A)** and corresponding DLP-printed ECM-MA constructs **(B)** demonstrate porous fibrillar architectures in both materials, with turquoise overlays indicating the pore segmentation masks used for quantitative analysis. Quantitative assessment revealed comparable two-dimensional porosity **(C)**, nearest-neighbor pore spacing **(D)**, and pore network connectivity assessed by the largest connected pore fraction (LCPF) **(E)** following methacrylation and DLP processing. Pore size distribution histograms based on maximum Feret diameter demonstrated bimodal pore populations in both materials **(F,G)**. Comparative analysis of mean pore size within each identified population showed a statistically significant but small magnitude difference in the lower pore size population, whereas the larger pore population did not differ significantly between decellularized ECM and ECM-MA constructs **(H)**. Quantification was performed on multiple independent and randomly selected regions of interest (n ≥ 5 per group). Data are presented as mean ± SEM. Statistical comparisons were conducted using unpaired two-tailed t-tests.

SEM was used to study the ultrastructural characteristics of the decellularized solid ECM matrix of the porcine endometrium compared to the 3D printed ECM-MA derived from it. Representative SEM micrographs revealed a porous fibrillar architecture in both the decellularized ECM and the printed ECM-MA constructs, with no overt qualitative differences in pore organization or interconnectivity (Figures 6A,B). Image segmentation enabled quantitative comparison of pore morphology and network features between the two materials (Figures 6C–H). Quantitative analysis (n ≥ 5 ROI per group) demonstrated comparable two-dimensional porosity between decellularized ECM and printed ECM-MA constructs (Figure 6C, 27.8% ± 5.3% and 30.9% ± 4.5%, respectively; p = 0.75). Similarly, nearest-neighbor pore distance did not differ significantly between conditions (Figure 6D, 0.64 ± 0.03 vs. 0.67 ± 0.04 µm; p = 0.79), and also the pore network connectivity, assessed by the largest connected pore fraction (LCPF), did not differ significantly following methacrylation and DLP printing (Figure 6E, 0.13 ± 0.04 vs. 0.18 ± 0.07, respectively; p = 0.29). Last, pore size analysis based on maximum Feret diameter revealed bimodal pore size distributions in both materials (Figures 6F,G). Two pore populations were identified. Comparative analysis of mean pore size within each population revealed a statistically significant but small-magnitude difference in the lower pore size population, whereas the larger pore population did not differ significantly between decellularized ECM and ECM-MA (Figure 6H). Together, these results indicate that the ultrastructural pore architecture of the native decellularized ECM is preserved following chemical modification and DLP-based fabrication.

### 3.4 Bioprinted dECM parenchymal cell support

**FIGURE 7:**
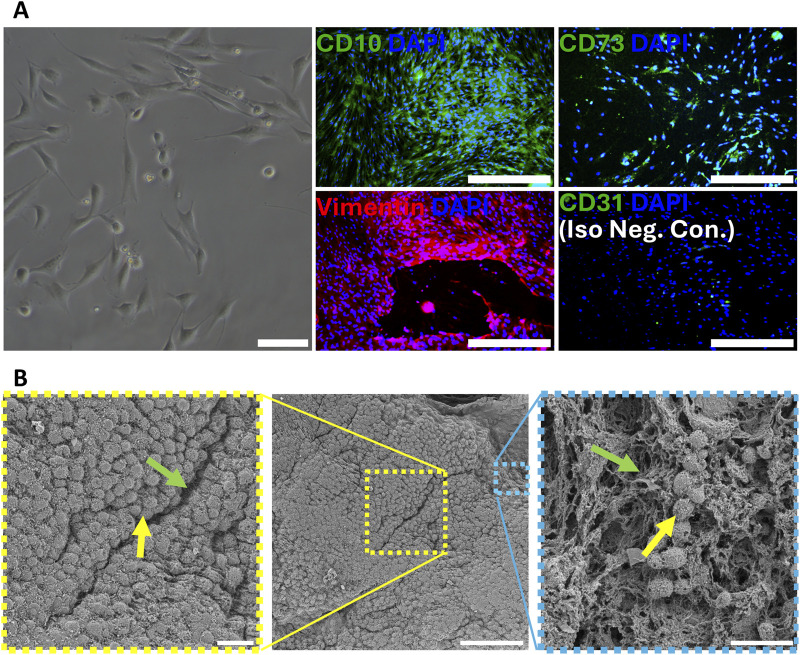
3D bioprinted dECM-MA bioink supports high-density parenchymal cell attachment, and early ECM remodeling. Human uterine stromal fibroblast cells (primary parenchymal cells of the uterine tissue, FC-0076 Lifeline Cell Technologies) were cultured in commercial media, exhibiting characteristic spindle-shaped stromal morphology **(A)**. These cells were immunofluorescently characterized for positive uterine and stromal markers (CD10, CD73, and Vimentin, respectively). CD31 isotype staining served as a negative control. Following expansion, cells were seeded at high density onto 3D bioprinted dECM-MA scaffolds, cultured for 16 h, and characterized by HR-SEM **(B)**. An overview SEM image shows a visibly high cell density on the ECM-MA after culture. Higher magnifications reveal a high-density (yellow rectangle, zoomed in) and lower density (blue rectangle, zoomed in) areas where human uterine stromal cells (yellow arrow) interact with the dECM-MA fibers (green arrow), suggesting biological interaction and constructive remodeling.

To evaluate the cell compatibility of the final printed constructs, and initial cell interactions we seeded human endometrial stromal fibroblasts (hESFs) onto 3D-printed scaffolds and cultured them for 16 h. Cells in monolayer culture exhibited characteristic spindle-shaped morphology (Figure 7A) and expressed stromal and endometrial markers including CD10, CD73, and vimentin, while CD31 served as a negative control. Scanning electron microscopy revealed widespread cell attachment to the dECM-MA scaffolds (Figure 7B, center), with both densely populated areas (Figure 7B, left) and sparser regions (Figure 7B, right) showing active cell–matrix interactions. Within the sparse regions the human uterine fibroblasts displayed well-spread morphologies and extended filopodia that interfaced intimately with ECM fibers, indicating favorable cell adhesion and early remodeling activity.

Together, these results demonstrate the feasibility of producing a standalone, DLP-compatible dECM-MA bioink from soft composite tissue. The resulting constructs preserve essential matrix composition and structure, support human stromal cell attachment, and are compatible with high-resolution 3D DLP bioprinting—highlighting their potential as biologically active scaffolds for engineered soft tissue applications.

## 4 Discussion

Standalone dECM bioinks remain scarce in 3D bioprinting due to inherent challenges with crosslinking efficiency, handling stability, and structural fidelity (Zhang H. et al., 2023). As a result, dECM-based bioinks, particularly in DLP, are typically formulated as hybrids, blending synthetic polymers such as gelatin methacryloyl (GelMA) or poly (ethylene glycol) diacrylate (PEGDA) to improve printability. In these systems, the polymeric network forms the primary crosslinked backbone, while the dECM component, even when methacrylated, contributes primarily to biochemical signaling cues and only limited mechanical reinforcement (Visscher et al., 2021; Zhang H. et al., 2023). Here, we present a photocrosslinkable dECM-MA bioink capable of DLP bioprinting without synthetic additives or additional polymeric reinforcement, which may otherwise dilute the bioactivity profile of the dECM in the final bioink formulation. Our results demonstrate that tissue-derived dECM bioinks can support stereolithographic biofabrication workflows as single-component systems while preserving key structural and compositional characteristics of the source material. By demonstrating that structurally and compositionally preserved dECM can be processed as a standalone material using DLP, this study establishes a fabrication framework that may be adapted to other soft-tissue–derived ECM sources. Because tissue-dependent bioactivity of dECM has been widely described in the literature (e.g., Zhang H. et al., 2023; Ramos-Rodriguez and Leach, 2025), adapting this fabrication strategy to other tissue sources may enable development of stereolithographically printed, tissue-associated bioinks in future studies.

Biochemical and histological characterization confirmed that our decellularization process effectively removed nuclear content (in accordance with established standards in this field (Crapo et al., 2011) while retaining collagen and to some extent also sulfated glycosaminoglycans (sGAGs). These findings are consistent with prior successful decellularization of uterine and soft tissues for regenerative medicine applications (De Vriendt et al., 2023; Sehic et al., 2024). The biochemical assessment performed here was intended to confirm decellularization efficiency based on established criteria, rather than to provide comprehensive proteomic characterization of all ECM components. TNBS quantification demonstrated a 40% reduction in accessible primary amine groups following methacrylation, indicating moderate functionalization of reactive lysine residues. This degree of substitution falls within the range commonly reported for methacrylated gelatin and ECM-derived biomaterials and has been associated with sufficient photocrosslinking capacity while maintaining native matrix characteristics (Pepelanova et al., 2018; Comperat et al., 2024). In this context, the observed degree of modification provides a chemically controlled basis for the subsequent DLP crosslinking and construct formation. Following methacrylation and formulation, the bioprinted constructs were self-supporting and suitable for manual handling and post-print processing. Quantitative printability analysis confirmed high geometric fidelity and controlled swelling comparable to PEGDA controls. We cautiously suggest that the sieve filtration step prior to enzymatic digestion may have contributed to the homogeneity of the ECM powder and enhanced the consistency of the liquefaction process. While we did not analyze molecular weight distributions directly, such filtration may help preserve longer protein fragments, potentially contributing to stable crosslinkable networks avoiding significant chain fragmentation (Gilbert et al., 2005).

Beyond biochemical composition, preservation of ECM microarchitecture is a critical determinant of scaffold function, yet it is rarely evaluated quantitatively in printed dECM systems. Our SEM and quantitative image analysis indicate that methacrylation and DLP printing largely preserve the ultrastructural pore architecture of the endometrial dECM at the scale resolved by SEM. No significant differences were observed in porosity, pore spacing, or network connectivity, and pore size distributions were overall comparable between printed ECM-MA constructs and the source decellularized solid ECM, with only a statistically significant but quantitatively minor shift in the lower pore size population (Figures 6A–H). This finding is notable in light of prior reports showing that enzymatic solubilization and reconstitution of bulk dECM into hydrogels commonly disrupt higher-order ECM organization, resulting in loss of dense collagen bundle architecture and a more loosened or randomized pore network, even when fibril-scale features remain comparable to the parent tissue (Pouliot et al., 2016). Similarly, in bulk cardiac dECM systems, digestion has been shown to increase pore size relative to native decellularized tissue, motivating the incorporation of reinforcing components to restore a more compact, tissue-like microarchitecture (Kong et al., 2023). In the context of DLP bioprinting, prior demonstrations of photocurable dECM have largely relied on polymer-reinforced hybrid formulations and have assessed microstructure primarily in a qualitative manner, without direct quantitative benchmarking against the source decellularized tissue (Ma et al., 2018). Against this background, the present results, to our knowledge, provide one of the first quantitative demonstrations that a standalone, DLP-printed methacrylated dECM construct can retain pore-level microarchitectural features comparable to its parent tissue. Together, these findings extend prior qualitative observations and support the structural fidelity of projection-based stereolithographic fabrication for soft-tissue dECM, demonstrating preservation of both biochemical identity and key microarchitectural features of the native dECM.

Two major photocrosslinking systems are widely used in 3D printing based biofabrication: the Ru/SPS system and the LAP–tartrazine system (Elkhoury et al., 2023; Lai et al., 2024). Both function within the visible spectrum (∼405–450 nm) and are compatible with light-based crosslinking. Ru/SPS initiates dityrosine bond formation and has been used in extrusion and volumetric printing of unmodified ECM and collagen due to its ability to crosslink without chemical modification (Steiner et al., 2025). LAP–tartrazine systems, by contrast, initiate free-radical polymerization of methacrylated moieties and have been broadly adopted in DLP bioprinting of materials such as PEGDA and hybrid dECM blends (Grigoryan et al., 2019; Zhang H. et al., 2023), where the polymer provides structural crosslinking and the dECM provides biochemical functionality. We evaluated both systems for our standalone dECM-MA bioink. Ru/SPS was tested for its compatibility with native ECM components, but ultimately failed to produce reproducible, self-supporting constructs under our DLP conditions. By contrast, LAP–tartrazine enabled reliable crosslinking of 8%–10% dECM-MA hydrogels with up to 30-s exposure times, producing a stable construct with consistent geometry and defined edges.

We did not experimentally dissect the mechanistic basis for the limited performance of Ru/SPS under our specific DLP parameters; however, several plausible factors may contribute. The poor performance of Ru/SPS in our DLP workflow may reflect differences in photoinitiation efficiency and crosslinking mechanism relative to LAP–tartrazine under 405 nm illumination. Although Ru/SPS is well-documented to initiate rapid crosslinking in bulk hydrogel systems—often faster than LAP—it is optimized for excitation near 450 nm, where its molar absorptivity is significantly higher (Yang et al., 2021; Elkhoury et al., 2023). At 405 nm, the reduced absorption of Ru(II) complexes may limit radical generation and subsequent network formation under DLP exposure conditions (Elkhoury et al., 2023). Additionally, Ru/SPS induces dityrosine bond formation via a redox-mediated mechanism (Ahmed et al., 2025; Steiner et al., 2025), whereas LAP–tartrazine drives free-radical polymerization of methacrylated residues (Elkhoury et al., 2023), which may be better aligned with methacrylated dECM systems, where photocrossliniking proceeds via free-radical vinyl conversion rather than protein side-chain oxidation. The inclusion of tartrazine also improves z-axis confinement by absorbing excess light and limiting overcuring beyond each layer (Grigoryan et al., 2019), thereby enhancing spatial fidelity. Furthermore, while Ru-SPS facilitates rapid polymerization in visible light bioprinting, including DLP (Li et al., 2021), its potential for oxygen inhibition in thin layers (Lai et al., 2024), particularly in the absence of effective photoabsorption, may be greater relative to LAP-tartrazine systems, where tartrazine may limit reaction volume and hence cause higher localized rate of oxygen depletion. Together, these factors may help explain the consistent success of LAP–tartrazine in our DLP bioprinting of dECM, and suggest that under the tested conditions Ru/SPS, while effective in bulk or volumetric photocrosslinking, may be less suited to thin-layer, 405 nm projection stereolithography using methacrylated bioinks.

In conclusion, our findings support the utility of LAP–tartrazine as a crosslinking system for standalone methacrylated dECM in DLP workflows. The constructs maintained high geometrical fidelity at millimeter scale, confirming the compatibility of this bioink with stereolithographic printing. Such a standalone dECM can provide tissue specific cues and bioactivity (Krishnamoorthi et al., 2020). Moreover, the process reported herein can be easily tuned for other soft tissues as well given the modularity of the different steps—each amenable to individual optimization—hence offering a practical workflow that can be potentially adapted toward a broader set of bioactive inks for DLP biofabrication. This platform, therefore, provides a foundation for high-resolution biofabrication of soft tissues, exemplified herein by a uterine model using chemically functionalized ECM, and advances the potential for physiologically and disease-relevant scaffold-driven tissue models in reproductive medicine and related disciplines. Direct incorporation of living cells into DLP-based formulations introduces additional variables related to light exposure, photoinitiator concentration, and potential phototoxicity, which were beyond the scope of the present material-processing study. Future work will explore integration of this formulation with cell-laden systems, and evaluate its long-term stability and cell-supporting ability.

## 5 Study limitations

While the present study establishes a reproducible DLP fabrication workflow for standalone dECM-MA bioinks and demonstrates dimensional fidelity, ultrastructural preservation, and early cell compatibility, comprehensive mechanical characterization was not performed. Quantitative assessment of compressive modulus, viscoelastic behavior, and crosslinking density will be important in future work to further define the mechanical performance of these constructs and to tailor stiffness for specific tissue applications. In addition, cell culture experiments in the present study were limited to short-term (16-h) evaluation of early attachment, while extended cytocompatibility analyses form part of a broader biological investigation underlying a separate manuscript and remain ongoing.

## Data Availability

The raw data supporting the conclusions of this article will be made available by the authors, without undue reservation.
