# Supplementary figures and images for: Standalone methacrylated extracellular matrix for digital light processing bioprinting: a practical workflow
Source: Front Bioeng Biotechnol. 2026 Apr 14;14:1774476. doi: 10.3389/fbioe.2026.1774476 (PMC13121313; doi:10.3389/fbioe.2026.1774476)

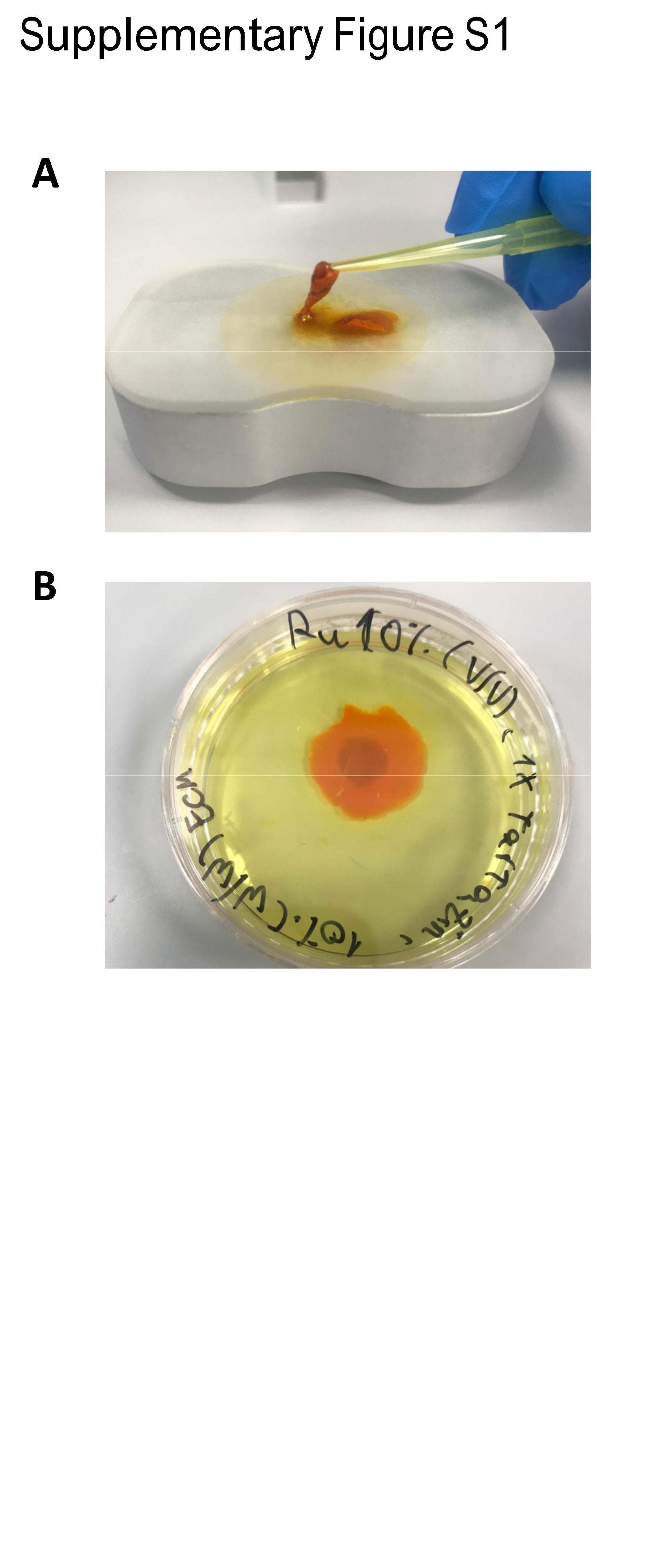

Supplement: Supplementary file 1 [file Image1.tiff]
