# Supplementary material for: Standalone methacrylated extracellular matrix for digital light processing bioprinting: a practical workflow
Source: Front Bioeng Biotechnol. 2026 Apr 14;14:1774476. doi: 10.3389/fbioe.2026.1774476 (PMC13121313; doi:10.3389/fbioe.2026.1774476)
Supplement: Supplementary file 2 [file Supplementaryfile1.docx]

**Supplementary Information**

**Methacrylated Extracellular Matrix for Digital Light Processing Bioprinting: A Practical Workflow**

Hod Bruck^1,†^, Shachar Sofer^1,†^, Aharon Lion^1,†^, Asher Ornoy^2,3^, Udi Sarig^1,2,^*

^1^ Department of Chemical Engineering, Faculty of Engineering, Ariel University, Ariel 4070000, Israel.

^2^ The Dr. Miriam and Sheldon Adelson School of Medicine, The Department of Morphological Sciences and Teratology, Ariel University, Ariel 4070000, Israel.

^3^ Department of Medical Neurobiology, Faculty of Medicine, The Hebrew University of Jerusalem, Jerusalem 9112102, Israel

^†^ These authors contributed equally to this work

* Corresponding author:

Dr. Udi Sarig (PhD)

Head—The Laboratory for Advanced Tissue Technologies (LATi-Tech)

Deputy Head—The Department of Medical Studies

Dr. Miriam and Sheldon Adelson School of Medicine,

Ariel University,

3 Kiryat Hamada Street,

Building 30, Room 6.14,

4070000 Ari'el,

Israel.

Telephone: +972-(0)3-6619 906

Mobile: +972-(0)58-402 6837

E-mail: [udis@ariel.ac.il](mailto:udis@ariel.ac.il)

Website: <https://www.ariel.ac.il/wp/lati-tech>

**Supplementary Figure S1:**

Representative images of ECM-MA based hydrogel constructs crosslinked using a Ru/SPS (10% v/v) photoinitiator system with tartrazine (1.5 mM) as a photoabsorber, printed with a 10% (w/w) ECM-MA formulation in a Lumen-X DLP bioprinter (BiCO, Sweden). Both samples were printed with a fixed layer exposure time of 30 seconds, while varying the light intensity.

**)A(** Underexposed sample printed at 30% intensity displays poor central gelation lacking fidelity to the printed design and minimal structure, consistent with sub-threshold activation of the Ru/SPS system. Remaining bioink can be observed on the inverted printing stage around the central printing area held by the pipette tip.

**(B**) Overexposed sample printed at 60% light intensity shows a dense, darkened core with a surrounding halo, indicative of excessive and non-specific crosslinking due to both local photoinitiator saturation and light scattering beyond the intended 4 mm cylindrical diameter pattern.

These results emphasize the challenge of fine-tuning the interplay between exposure time, light intensity, photoinitiator concentration, and photoabsorber concentration in this system. The narrow operational window of Ru/SPS with ECM-MA based hydrogels makes it particularly sensitive to small parameter shifts, leading to either over-polymerization and loss of spatial resolution, or under-crosslinking and incomplete structure formation. Careful optimization and potentially dynamic exposure control are required for reproducible and high-fidelity printing.
